# Silkworm Coatomers and Their Role in Tube Expansion of Posterior Silkgland

**DOI:** 10.1371/journal.pone.0013252

**Published:** 2010-10-12

**Authors:** Qiao Wang, Birong Shen, Pengli Zheng, Hui Feng, Liang Chen, Jing Zhang, Chuanxi Zhang, Guozheng Zhang, Junlin Teng, Jianguo Chen

**Affiliations:** 1 The Key Laboratory of Cell Proliferation and Differentiation of Ministry of Education, The State Key Laboratory of Bio-membrane and Membrane Bio-engineering, College of Life Sciences, Peking University, Beijing, China; 2 The Center for Theoretical Biology, Peking University, Beijing, China; 3 Institute of Insect Sciences, Zhejiang University, Zhejiang, China; 4 The Sericultural Research Institute, Chinese Academy of Agricultural Sciences, Zhejiang, China; Tufts University, United States of America

## Abstract

**Background:**

Coat protein complex I (COPI) vesicles, coated by seven coatomer subunits, are mainly responsible for Golgi-to-ER transport. Silkworm posterior silkgland (PSG), a highly differentiated secretory tissue, secretes fibroin for silk production, but many physiological processes in the PSG cells await further investigation.

**Methodology/Principal Findings:**

Here, to investigate the role of silkworm COPI, we cloned six silkworm COPI subunits (α,β,β′, δ, ε, and ζ-COP), determined their peak expression in day 2 in fifth-instar PSG, and visualized the localization of COPI, as a coat complex, with *cis*-Golgi. By dsRNA injection into silkworm larvae, we suppressed the expression of α-, β′- and γ-COP, and demonstrated that COPI subunits were required for PSG tube expansion. Knockdown of α-COP disrupted the integrity of Golgi apparatus and led to a narrower glandular lumen of the PSG, suggesting that silkworm COPI is essential for PSG tube expansion.

**Conclusions/Significance:**

The initial characterization reveals the essential roles of silkworm COPI in PSG. Although silkworm COPI resembles the previously characterized coatomers in other organisms, some surprising findings require further investigation. Therefore, our results suggest the silkworm as a model for studying intracellular transport, and would facilitate the establishment of silkworm PSG as an efficient bioreactor.

## Introduction

The silkworm, *Bombyx mori*, produces silk, and is considered as one of the best-characterized biological model organisms [1], [2]. Because of the economic significance and benefit of silk production, the composition and secretion of silk has been intensively investigated. Fibroin, one of the major silk components, is composed of heavy chain proteins, light chain proteins, and P25, and is secreted by the posterior silkgland (PSG) [3], [4]. The fibroin-containing vesicles are potentially transported by BmKinesin-1 from the Golgi apparatus to the apical cytoplasm, and finally the fibroin is released into the glandular lumen [5]. In addition, silkworm has been proposed to be a potential “bioreactor” for biotechnology and pharmacy [1], [6], [7]. PSG cells devote 85% of their protein synthesis to silk production [8]. Recombinant human procollagen has been successfully expressed in the PSG by transgenesis, raising the possibility of efficiently and abundantly expressing pharmaceutical proteins in this system [6]. To achieve this, it is necessary to understand the molecular mechanisms of various intracellular transport processes in PSG, including fibroin secretion, which are still largely elusive.

Eukaryotic cells possess an elaborate endomembrane system, which is responsible for protein biogenesis. In this system, various membrane-enclosed organelles communicate with each other through vesicular transport. These coated vesicles, generated at donor compartments and then fused with the destination compartments, are enveloped by distinct sets of coats and are responsible for highly selective transport [9]–[12]. They can mainly be classified into clathrin-coated vesicles and non-clathrin-coated vesicles [13], [14]. Coat protein complex I (COPI) vesicles belong to the non-clathrin-coated vesicles, and are well-characterized for retrogradely delivering proteins from the Golgi apparatus to the endoplasmic reticulum (ER) [9], [10], [15].

The COPI complex is composed of coatomer subunits [16], named α- (160 kDa), β- (107 kDa), β′- (102 kDa), δ- (60 kDa), ε- (36 kDa), γ- (97 kDa), and ζ-COP (20 kDa). Based on the sequential and structural similarities with adaptor protein 2 (AP2) complexes, the structure of the COPI heptamer is predicted to have an inner subcomplex (βδγζ) and an outer subcomplex (αβ′ ε) [17], [18]. In higher eukaryotes, there exist two versions of γ- and ζ-COP: γ1, γ2, ζ1, and ζ2, which form three main types of heptamer γ1ζ1,γ1ζ2,and γ2ζ1 [19]. Significant localization differences for these COPI-isoforms were found [20], suggesting that different COPI isoforms carry out distinct physiological functions.

COPI vesicles are best characterized as carriers participating in retrograde transport from *cis*-Golgi back to ER [21], [22]. However, the COPI vesicles were originally reported to be involved in intra-Golgi transport [13], though the mechanism has long been in debate [12], [23]. COPI is also reported to be associated with the endosomal activities, endocytosis, and autophagy [24], [25]. During transport, cargo proteins with sorting signals can be recognized and further captured into COPI-coated vesicles [9], [22]. Coatomer subunits in different organisms, including yeast, plants, insects, and mammals, have been cloned and demonstrated to be highly conserved [10]. However, the role of COPI in some specific tissues, such as the silkworm PSG, is yet to be studied.

Here, we cloned six silkworm coatomers, including α-, β-, β′-, δ-, ε-, and ζ-COP, and selected the RNA interference (RNAi) technique, which is well documented in fly and silkworm [26]–[28], to investigate the physiological functions of COPI in the silkworm PSG. We found that COPI deficiency disrupts the Golgi apparatus and causes narrower PSG glandular lumen, indicating that COPI is essential for the integrity of endomembrane system in PSG cells and for PSG tube expansion.

## Results

### Cloning of silkworm coatomer subunits

From yeast to mammals, the COPI complex is composed of seven coatomer subunits. However, only the γ-COP sequence has been reported in silkworm [29]. Therefore, we cloned the other six silkworm coatomers. The reported coatomer sequences in various organisms share a high sequence similarity, especially within several conserved domains [10], [30]. Based on the sequence conservation between the silkworm and its evolutionary partner *Drosophila melanogaster*, we searched for coatomers in the silkworm genome [31] and cDNA databases [32] using Basic Local Alignment Search Tool (BLAST). The entire nucleotide sequences of silkworm α-, β-, β′-, δ-, ε-, and ζ-COP subunits, including the start codon, the stop codon, the 5′-untranslated region (UTR), and the 3′-UTR, were successfully predicted. Based on these predictions, we further designed primers and amplified the six subunits from silkworm PSG cDNA by reverse transcription PCR (RT-PCR). We named these six coatomer subunits Bm-α-COP (BmCOPA), Bm-β-COP (BmCOPB), Bm-β′-COP (BmCOPB2), Bm-δ-COP (BmCOPD), Bm-ε-COP (BmCOPE), and Bm-ζ-COP (BmCOPZ) (GenBank Accession Numbers: **GU322815 - GU322820**) (Table S1).

### Bioinformatic analysis

**Figure 1:**
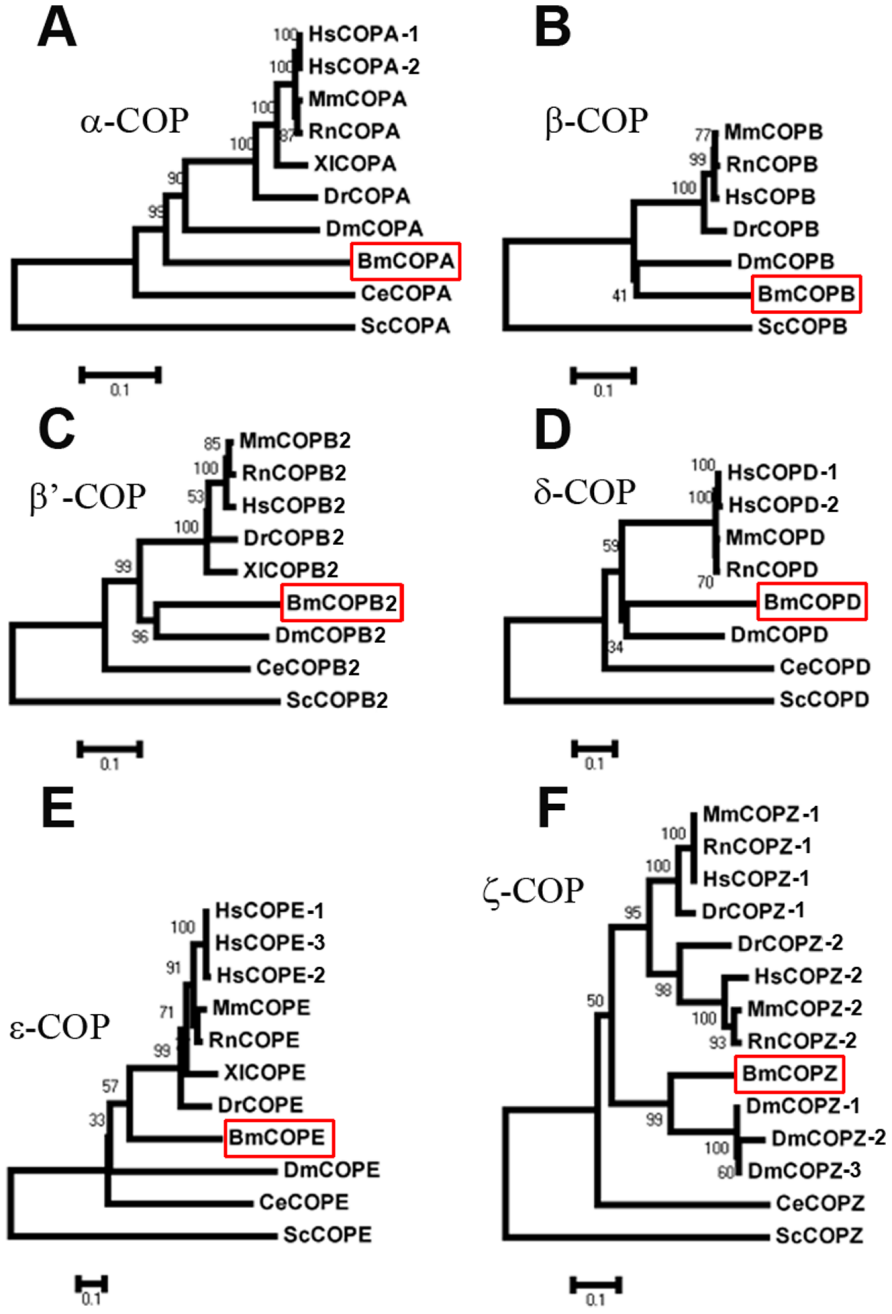
Phylogenic trees of six silkworm coatomer subunits. Six phylogenic trees of α-COP (COPA) (A), β-COP (COPB) (B), β′-COP (COPB2) (C), δ-COP (COPD) (D), ε-COP (COPE) (E), and ζ-COP (COPZ) (F) are shown. Each tree contains several coatomer homologues from different organisms and is arbitrarily rooted using coatomers from *S. cerevisiae*. The red borders indicate the silkworm coatomer subunits. Bm, *Bombyx mori*; Ce, *Caenorhabditis elegans*; Dm, *Drosophila melanogaster*; Dr, *Danio rerio*; Hs, *Homo sapiens*; Mm, *Mus musculus*; Rn, *Rattus norvegicus*; and Sc, *Saccharomyces cerevisiae*.

We deduced the amino acid sequences of the six coatomers from complete cDNA sequences. Bm-α-COP contains 1230 amino acids with a predicted molecular mass of 138 kDa, Bm-β-COP has 573 amino acids and is 64 kDa, Bm-β′-COP has 935 amino acids and is 105 kDa, Bm-δ-COP has 507 amino acids and is 56 kDa, Bm-ε-COP has 302 amino acids and is 33 kDa, Bm-ζ-COP has 183 amino acids and is 20 kDa. To systematically analyze the coatomers, we examined the sequence identity of the six coatomers between silkworm and other organisms (Table S1), and further demonstrated that the coatomer sequences were highly conserved throughout evolution (Figure 1). The sequence identities of Bm-α-COP were 62% with its homologues in *Drosophila*, 60% with human, and 35% with *S. cerevisiae* (Table S1). However, it is worth mentioning that identified silkworm β-COP lacks nearly 400 amino acids in the middle region compared with its homologues in other organisms. We speculate that this silkworm β-COP is an isoform produced by alternative splicing. However, as far as we know, no isoform of β-COP has been reported previously in other organisms. Therefore, to identify the β-COP with the 400 amino acids in silkworm, or to look for potential β-COP splicing variants in mammals, will extend the information about coatomer isoforms and different COPI heptamers.

We then constructed phylogenic trees for each coatomer, all of which were arbitrarily rooted using coatomers from *S. cerevisiae* (Figure 1). Both phylogenic trees and sequence alignments demonstrated the sequence conservation of coatomer subunits between silkworm and other organisms, especially between silkworm and *Drosophila*. This evolutionary conservation further suggests similar roles of silkworm coatomers as coat proteins during intracellular transport.

### Tissue distribution during developmental stages

**Figure 2:**
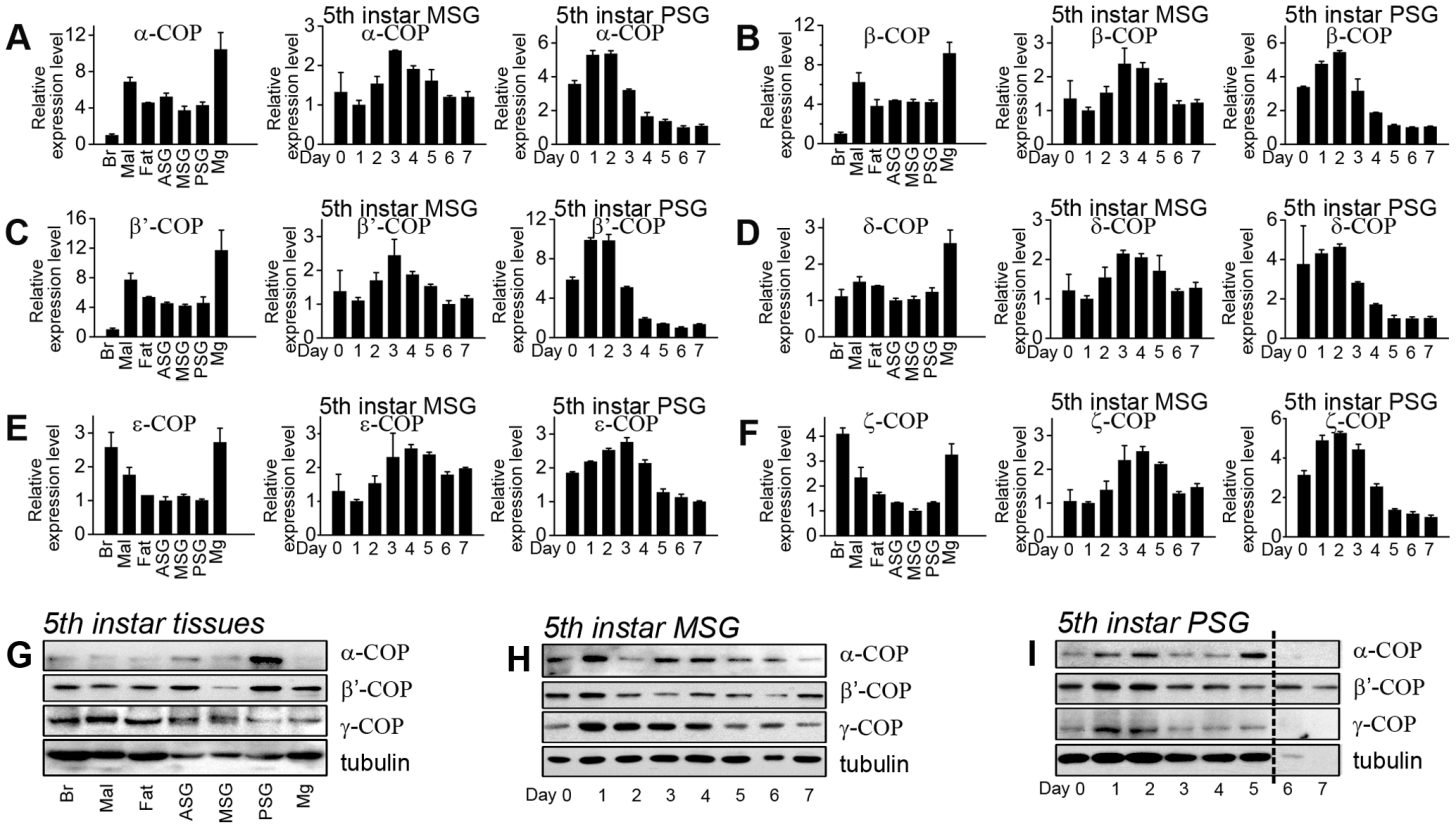
Expression patterns of six silkworm coatomers. (A–F) Relative mRNA expression patterns of Bm-α-COP (A), Bm-β-COP (B), Bm-β′-COP (C), Bm-δ-COP (D), Bm-ε-COP (E), and Bm-ζ-COP (F). The three bar graphs from left to right are respectively the relative mRNA expression levels in seven different tissues of silkworm, in eight different days (Day 0–7) of the fifth-instar MSG, and in eight different days of the fifth-instar PSG. (G–I) Protein expression levels of Bm-α-COP, Bm-β′-COP, and Bm-γ-COP were detected in different tissues (G), the fifth-instar developmental MSG (H), and the fifth-instar developmental PSG (I). The seven tissues are Brain (Br), Malpighian tubule (Mal), fat body (Fat), anterior silkgland (ASG), middle silkgland (MSG), posterior silkgland (PSG), and midgut (Mg). Day 0–7 indicates day 0–7 of the fifth-instar larvae.

We performed relative real-time PCR analysis to examine the relative expression levels of the six coatomers in different tissues during different developmental stages. *Ribosomal protein L3* (*RpL3*), which exhibits the most ubiquitous expression in silkworm [33], was used as a quantity reference. The mRNA transcripts of all the six coatomer subunits were detected in brain, Malpighian tubule, fat body, anterior silkgland (ASG), middle silkgland (MSG), PSG, and midgut of fifth-instar day-3 larvae, with the highest expression in midgut (Figure 2A–F, left). Their expression patterns in the tissues were nearly the same, except that the α, β, and β′ subunits exhibited lower levels in the brain (Figure 2A–C, left), whereas the transcripts of δ, ε, and ζ subunits were enriched in the brain (Figure 2D–F, left). This variation implies that δ, ε, and ζ subunits have specific physiological functions in silkworm brain.

To determine the mRNA changes in different developmental stages, we collected the MSGs and PSGs from fifth-instar larvae from day 0 (shortly after the fourth ecdysis) to day 7 (one day before cocooning), and performed real-time PCR. During this stage, the expression patterns of all six coatomers in the MSG were similar: increasing during the first three days, reaching a peak at days 3–4, and then decreasing slightly (Figure 2A–F, middle). In the PSG, the transcripts of these coatomers accumulated during the first three days, but exhibited a similar trend of decrease in the following five days (Figure 2A–F, right).

Next, to examine the protein expression patterns of COPI subunits, we produced rabbit and mouse polyclonal antibodies (Figure S1). These coatomer proteins were abundant in PSG, but lower in the brain using tubulin as a reference (Figure 2G). In the MSG, α-, β′-, and γ-COP proteins maintained a relatively stable expression level but fluctuated slightly from day to day (Figure 2H). In the PSG, COPI coatomer proteins increased and reached a peak during the first three days (Figure 2I). Besides, we observed an evident degradation of tubulin in PSGs from days 6–7 (Figure 2I), which may be due to programmed cell death caused by pupal metamorphosis [2], [34], [35].

### Subcellular distribution of silkworm COPI

**Figure 3:**
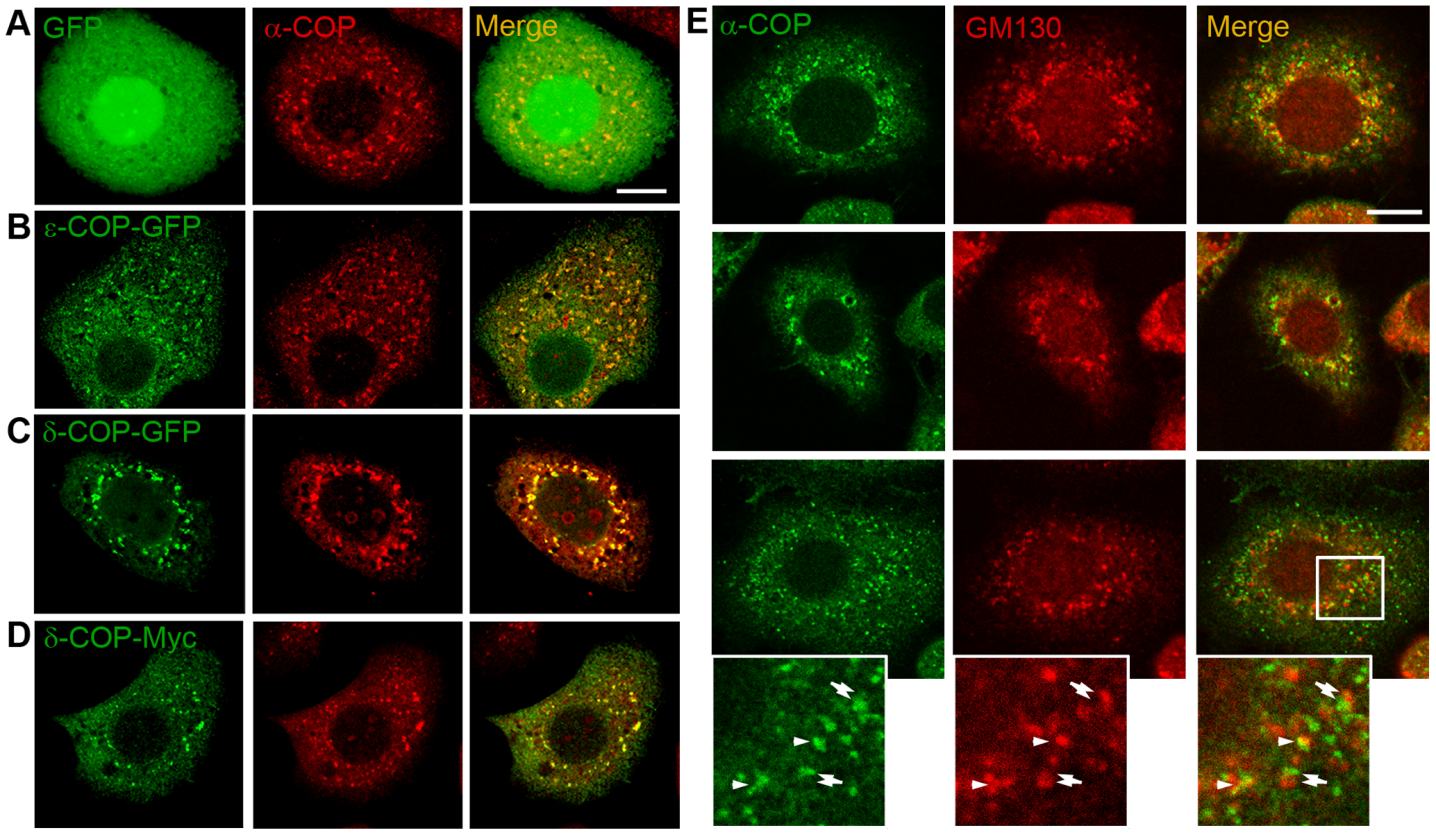
Subcellular distribution of silkworm COPI. (A–D) The ε-COP-GFP, δ-COP-GFP and δ-COP-Myc colocalized with endogenous α-COP in BmN cells. The BmN cells transfected with GFP (A, green), or ε-COP-GFP (B, green), or δ-COP-GFP (C, green) were immunolabeled with anti-α-COP antibody (red). (D) BmN cells transfected with δ-COP-Myc were co-immunostained with anti-Myc (green) and anti-α-COP (red) antibodies. (E) Subcellular distribution of α-COP in BmN cells. BmN cells were co-immunostained with anti-α-COP antibody (green) and anti-GM130 antibody (red). Three different cells are presented separately, and the rectangle is magnified in the left lower panel. Arrowheads point to colocalized dots, while double arrows indicate adjacent green/red dots. Scale bars represent 10 µm.

Previous biochemical and kinetic analysis shows that the fusion protein ε-COP-GFP can be stably assembled into coatomer complexes [36]. To test the immunofluorescence staining efficiency of the anti-Bm-α-COP antibody, we overexpressed Bm-ε-COP-GFP in BmN cells under the hr5-enhancer/IE-1 promoter [37], [38], and immunolabeled the transfected BmN cells with this antibody. Endogenous immunolabeled α-COP (Figure 3A–B, red) localized in a manner indistinguishable from that of ε-COP-GFP (Figure 3B, green), showing that the produced anti-Bm-α-COP antibody efficiently labeled the endogenous COPI complex. To further confirm this result, we overexpressed δ-COP-Myc and δ-COP-GFP in BmN cells. The subcellular distributions of δ-COP-GFP (Figure 3C, green) and δ-COP-Myc (Figure 3D, green) were also nearly the same as that of endogenous α-COP (Figure 3C–D, red). Therefore, the anti-Bm-α-COP antibody can effectively label the endogenous silkworm COPI complex.

**Figure 4:**
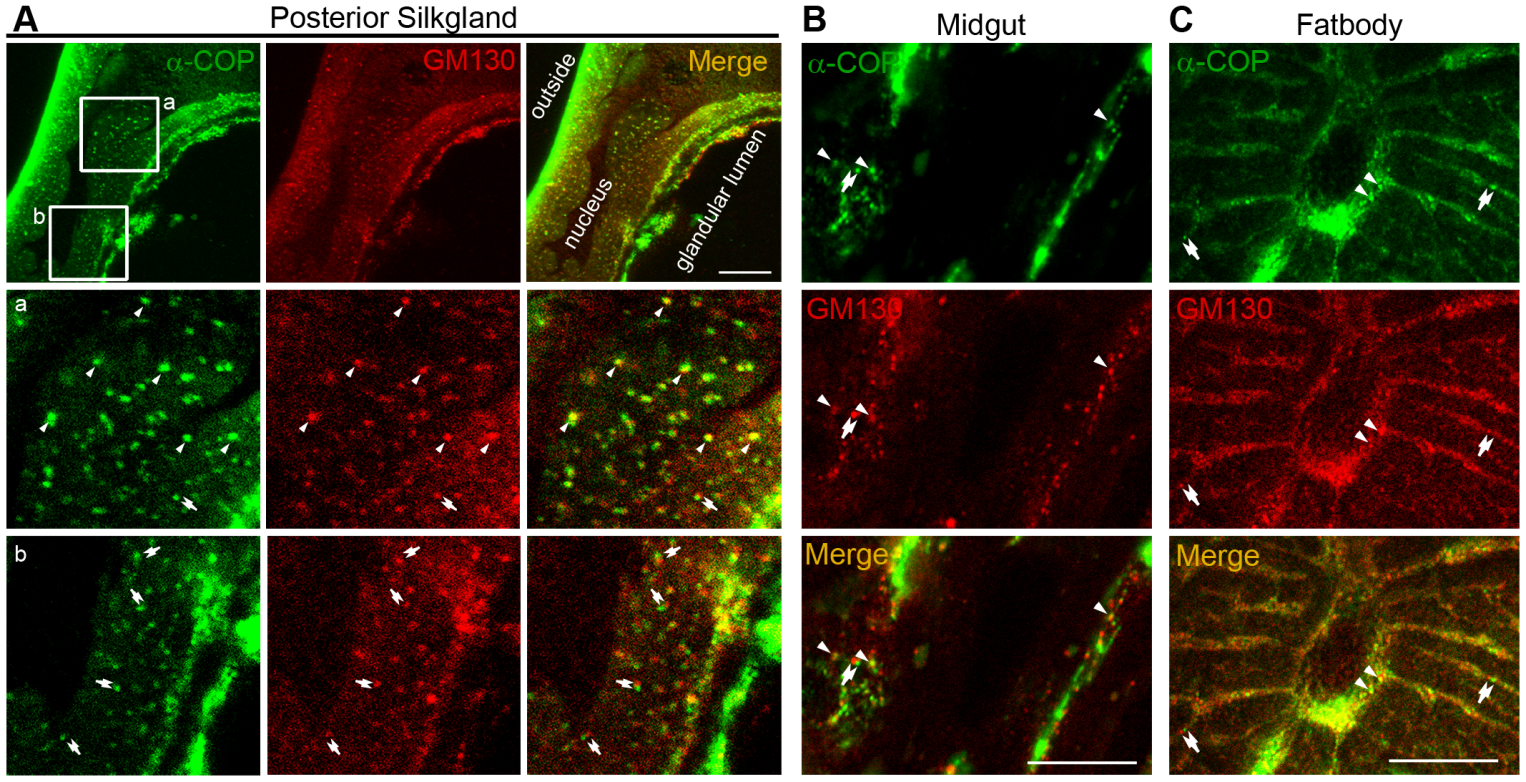
α-COP partially colocalized with *cis*-Golgi in PSG, midgut and fat body. (A–C) Cryosections of PSG (A), midgut (B) and fat body(C) co-immunostained with anti-α-COP antibody (green) and anti-GM130 antibody (red). Boxed areas ‘a’ and ‘b’ are magnified. The colocalized green and red dots are indicated by arrowheads; and adjacent dots are indicated by double arrows. Scale bars represent 10 µm.

We further used this anti-Bm-α-COP polyclonal antibody to trace the native subcellular localization of the COPI complex in BmN cells. As shown, in BmN cells, COPI complexes were enriched and distributed throughout the cytoplasm (Figure 3E). Co-immunostaining of α-COP with the *cis*-Golgi marker GM130 [39] showed that a significant proportion of Bm-α-COP dots colocalized with or localized adjacently to *cis*-Golgi in BmN cells (Figure 3E). Indirect immunofluorescence of PSG cryosections further demonstrated the partial overlapping of Bm-α-COP with *cis*-Golgi protein GM130 (Figure 4A). In the high magnification figures of both BmN cells and PSG cryosections, some COPI complexes colocalized with the *cis*-Golgi, while others localized adjacent to the *cis*-Golgi marker GM130 (red) (Figure 3E and 4A). The findings in silkworm fat body and midgut were the same (Figure 4B–C). In normal rat kidney (NRK) cells, γ1-, and ζ2-COPs are reported to localize at the *cis* half of the Golgi apparatus; the γ2-COPs are mostly restricted at *trans*-Golgi; and the endogenous β′-COPs are observed throughout the whole Golgi areas [20]. In *Drosophila*, a discrete colocalization of γ-COP with *cis*-Golgi and ER was reported, but no colocalization with *median*- or *trans*-Golgi markers [40]. Our results are consistent with previous reports, and demonstrate that silkworm COPI is also partially colocalized with *cis*-Golgi (Figure 3E and 4).

**Figure 5:**
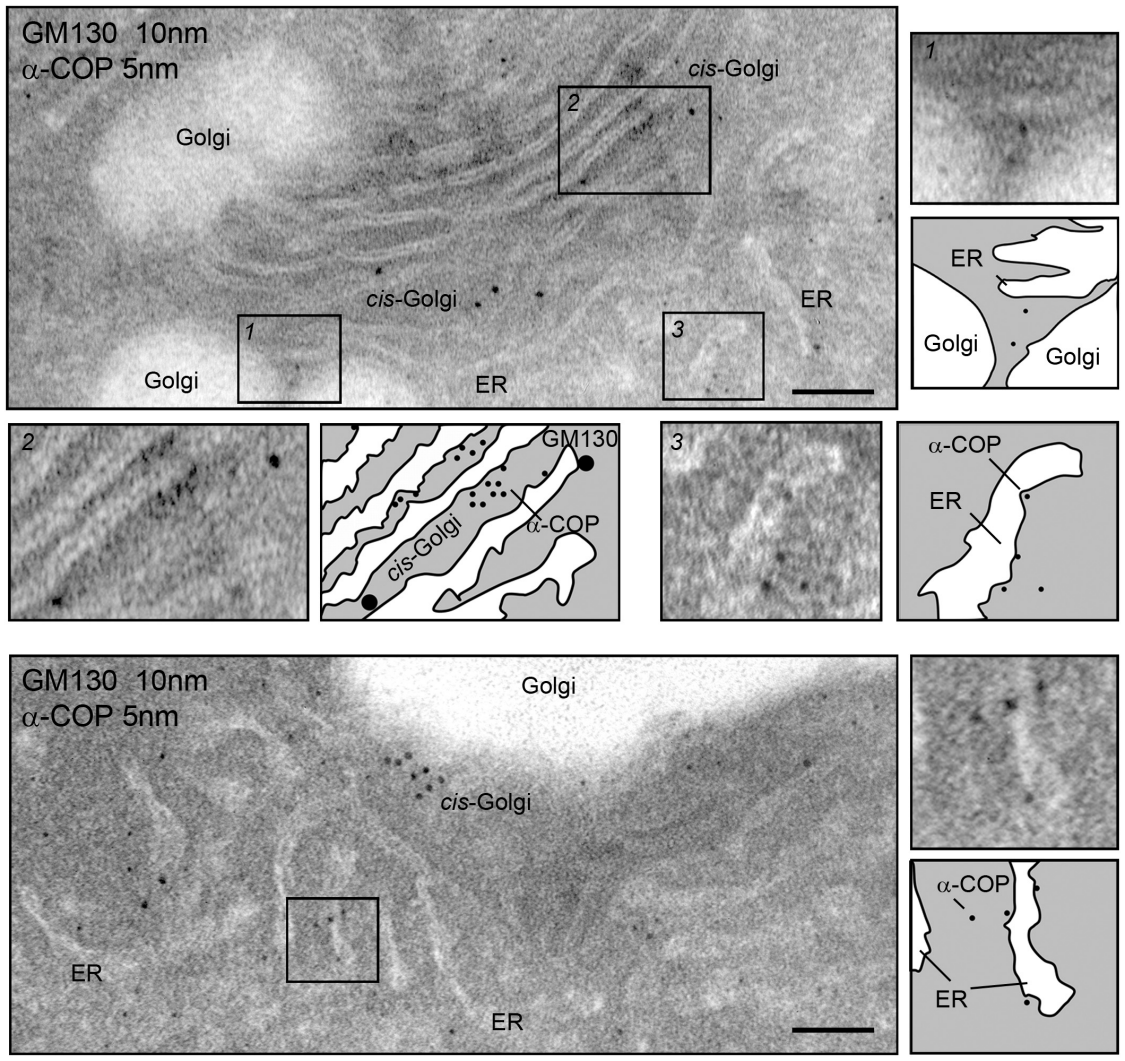
Immunoelectron microscopy of α-COP in PSG cells. Immunogold labeling of PSGs. Representative electron micrographs of endomembrane system double-labeled with anti-α-COP (bound to 5-nm protein A-gold) and anti-GM130 antibodies (bound to 10-nm protein A-gold). The rectangles are magnified, along with schematic diagrams. Scale bars represent 100 nm.

To better characterize the silkworm COPI complex, we detected the distribution of COPI using immunogold electron microscopy (EM) in PSG cells with mouse anti-Bm-α-COP and rabbit anti-GM130 (*cis*-Golgi marker) antibodies (Figure 5). Immunogold labeling showed that gold-labeled Bm-α-COPs (5 nm) resided in the cytosol and accumulated around the *cis*-Golgi marker GM130 (10 nm). Some Bm-α-COPs were also attached to the cytosolic side of the Golgi apparatus and ER (Figure 5). These results indicate that silkworm coatomers act as vesicle coats participating in intracellular transport between different cellular compartments.

### COPI is required for PSG tube expansion

**Figure 6:**
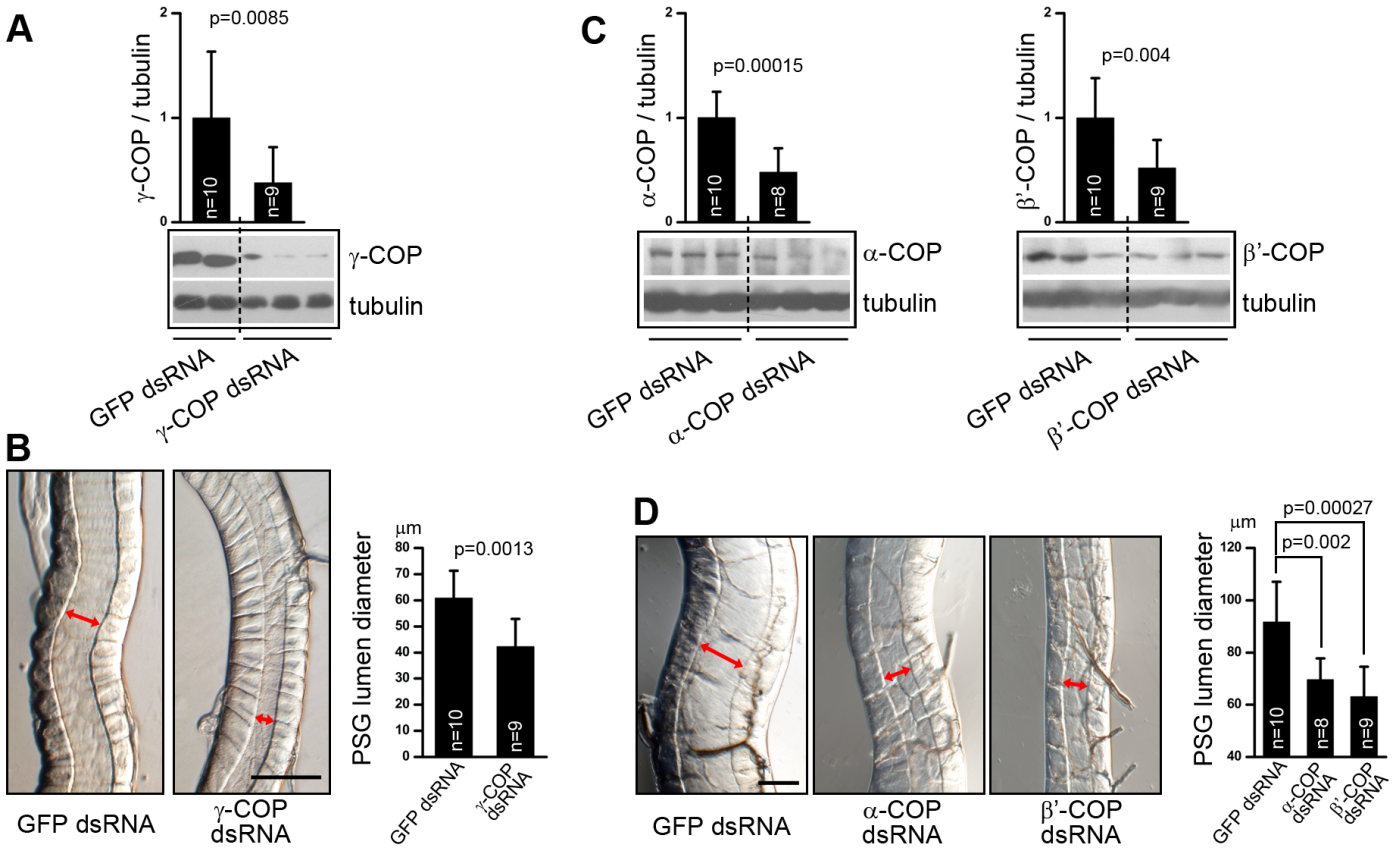
COPI is required for PSG tube expansion. (A) Western blotting of second-instar silkworm larvae injected with GFP dsRNA (left two lanes) or γ-COP dsRNA (right three lanes). Relative protein expression levels of γ-COP, with tubulin as a reference control, are shown as the column. (B) Second-instar silkworm PSG microphotographs, with arrows indicating the glandular luminal diameter. Calculated diameters of PSG lumen in silkworms injected with GFP or γ-COP dsRNAs are shown in the right bar graph. (C) Western blotting shows suppressed expression of the α-COP (left) and β′-COP (right) by RNAi in third-instar silkworm larvae. Relative protein expression levels of α- and β′-COP, with tubulin as a reference control, are shown. (D) Silkworm PSG microphotographs in third-instar silkworms injected with GFP, α-COP, or β′-COP dsRNAs. Arrows indicate glandular luminal diameter. Statistical calculation of PSG luminal diameters are shown in the right bar graph. Scale bars represent 100 µm.

To test our hypothesis that silkworm COPI is involved in PSG development, we knocked down the expression level of previously identified γ-COP [29] (Table S1) and examined its effect on PSG morphology. We injected γ-COP dsRNA (nucleotide 751–1500 base pairs) and GFP dsRNA (as a control) into the second-instar larvae. Five days later, we assessed the expression level of native γ-COP using the mouse anti-γ-COP antibody (Figure S1). The silkworms injected with γ-COP dsRNA exhibited a significant reduction on native γ-COP protein, to a level of ∼40% in those silkworms injected with GFP dsRNA (Figure 6A). Meanwhile, we found that the down-regulation of γ-COP led to a narrower PSG glandular lumen (Figure 6B). The average diameter of the glandular lumen in silkworms injected with γ-COP-dsRNA decreased from 60 µm to 42 µm (Figure 6B), showing that knockdown of γ-COP affected PSG tube expansion.

Accordingly, we speculated that the COPI complex, which contains γ-COP as one of its seven subunits, is required for PSG tube expansion. To examine whether knockdown of α- and β′-COP repress PSG tube expansion like γ-COP, the synthesized dsRNAs of α- (nucleotide 13–660 and 3061–3720 base pairs), β′-COP (nucleotide 1–660 and 661–1200 base pairs), and GFP were injected into third-instar larvae (Figure 6C–D). In the silkworms injected with α-COP dsRNA, a statistically significant reduction (∼48%) of α-COP expression level was observed five days later (Figure 6C, left). The PSG luminal diameter in these silkworms was significantly narrower (Figure 6D), averaging approximately 69 µm, compared with 91 µm in controls. In the silkworms injected with β′-COP dsRNA, the result was similar: ∼52% reduction of β′-COP protein (Figure 6C, right) led to ∼31% decrease of average PSG luminal diameter (Figure 6D). Furthermore, it is worth mentioning that the knockdown of COPI subunits in both second- and third-instar larvae suppressed the diameter increase of the PSG glandular lumen, implying that COPI is essential for PSG tube expansion throughout the larval stage. Moreover, during the first three days of the fifth-instar larval stage, the PSG develops very rapidly, and the diameter of the PSG increases dramatically [41]. During that period, most coatomer subunits are expressed at elevated levels in PSG cells, also suggesting that COPI is associated with PSG growth during the fifth-instar larval stage (Figure 2). Taken together, these findings that a narrower PSG lumen corresponds to lower coatomer expression levels suggest that silkworm COPI is required for the tube expansion of PSG.

### COPI deficiency disrupts the integrity of Golgi apparatus

**Figure 7:**
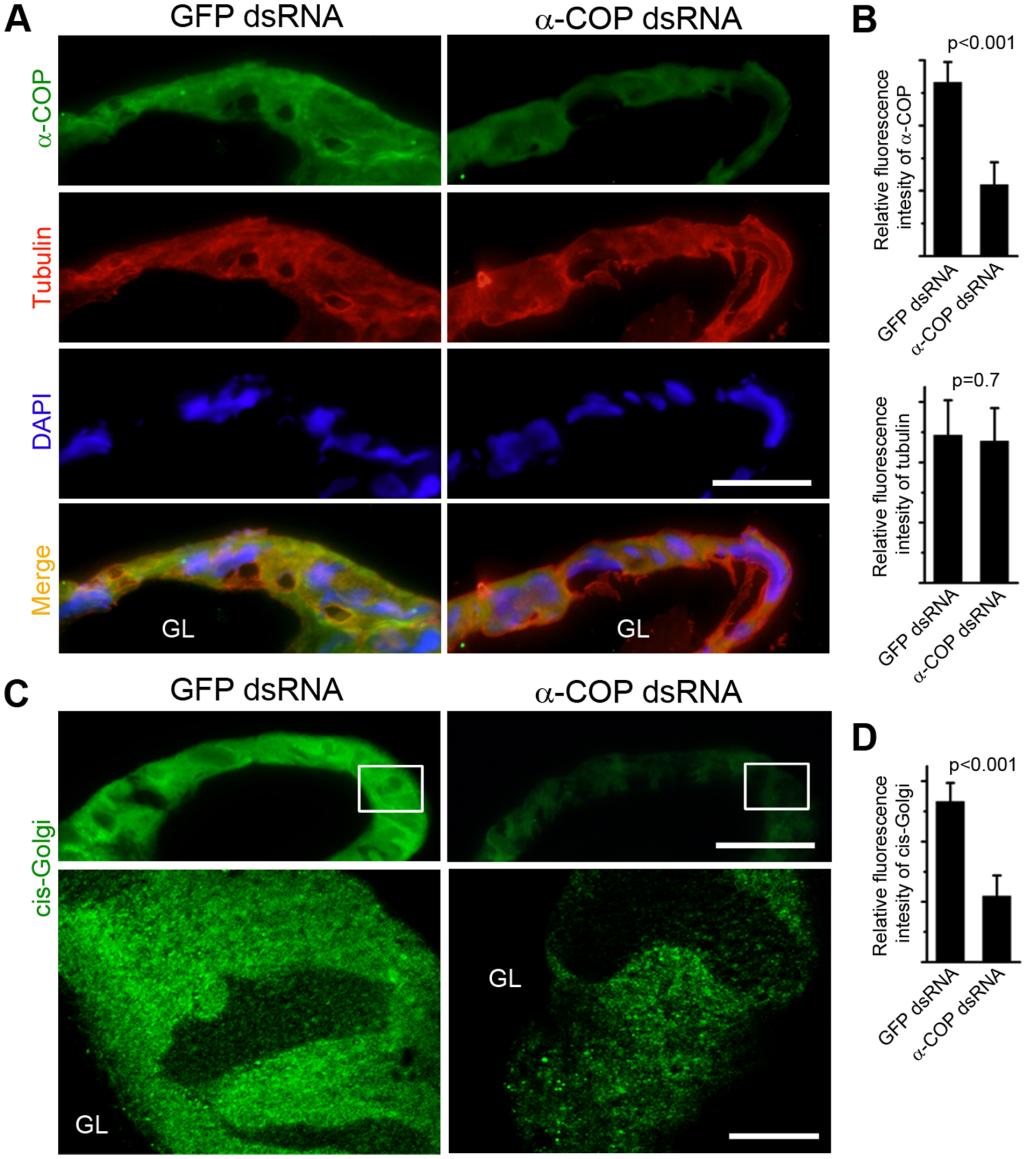
Immunohistofluorescence of dsRNA-treated PSG cryosections. (A) Cryosections of PSGs from GFP/α-COP dsRNA injected silkworms are co-immunostained with anti-α-COP antibody (green) and anti-tubulin antibody (red). DAPI stains nuclei. Scale bar represents 50 µm. (B) Relative fluorescence intensities of α-COP and tubulin in (A) are shown. (C) Cryosections of PSGs from GFP/α-COP dsRNA injected silkworms are immunostained with anti-GM130 antibody. The rectangles in the upper panel (scale bar represents 50 µm) are magnified in the lower panel (scale bar represents 10 µm). (D) Relative fluorescence intensity of GM130 in (C) is shown. GL represents glandular lumen.

To elucidate the underlying mechanism of tube expansion failure in COPI-deficient PSG, we first stained the dsRNA-treated PSG cryostat sections. The relative fluorescence intensity of α-COP was dramatically reduced after α-COP dsRNA treatment, while the fluorescence intensity of tubulin did not change (Figure 7A–B), which were consistent with the Western blotting (Figure 6C). We further examined the *cis*-Golgi apparatus by immunolabeling GM130. In wildtype PSG cells, the GM130 exhibited a punctate cytoplasmic signal (Figure 4A). However, α-COP dsRNA injection decreased the punctate staining of GM130, compared with the GFP dsRNA-treated samples (Figure 7C). Statistic data showed a drastic reduction in the staining intensity of GM130 in the α-COP knockdown PSGs compared with the control PSGs (Figure 7C–D). These data suggest that the knockdown of α-COP in PSG causes the structural defects of Golgi apparatus.

**Figure 8:**
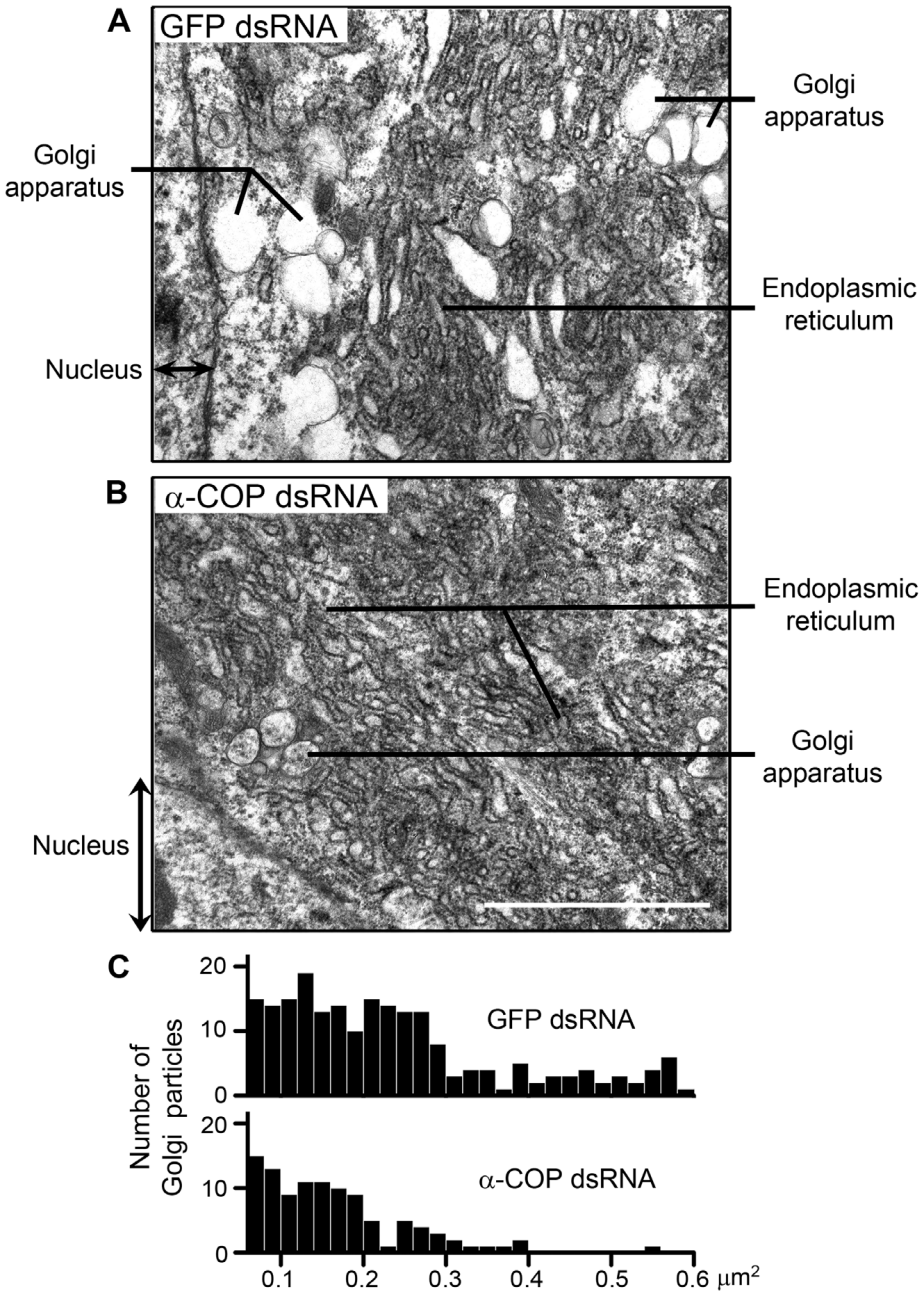
Detailed subcellular structures of COPI-deficient PSG. (A) A representative electron micrograph of PSGs from GFP dsRNA-injected silkworms. (B) A representative electron micrograph of PSGs from α-COP dsRNA-injected silkworms. Scale bars represent 500 nm. (C) Statistic graphs of number (Y coordinate) and area (X coordinate) of Golgi particles in GFP/α-COP dsRNA-injected silkworms, according to the electron micrographs.

To further confirm our results, we observed the detailed subcellular structure in dsRNA-treated PSG by electron microscopy. The electron micrographs showed abundant Golgi apparatus and ER in the fourth-instar PSG cells (Figure 8A). In contrast to the numerous and apparent Golgi particles and ER network, the endomembrane system in the α-COP knockdown PSGs appeared indistinct and disordered. Especially, the bubble-like Golgi structure after α-COP dsRNA treatment became less and fragmentated (Figure 8A–B). We calculated the number and area of Golgi particles, and further demonstrated that knockdown of α-COP decreased the population and volume of Golgi apparatus in PSG cells (Figure 8C). Taken together, in α-COP deficient PSGs, the integrity of endomembrane system, especially the structure of Golgi apparatus, is disrupted, suggesting that the defect in tube expansion may be due to the collapsed endomembrane system.

## Discussion

**Figure 9:**
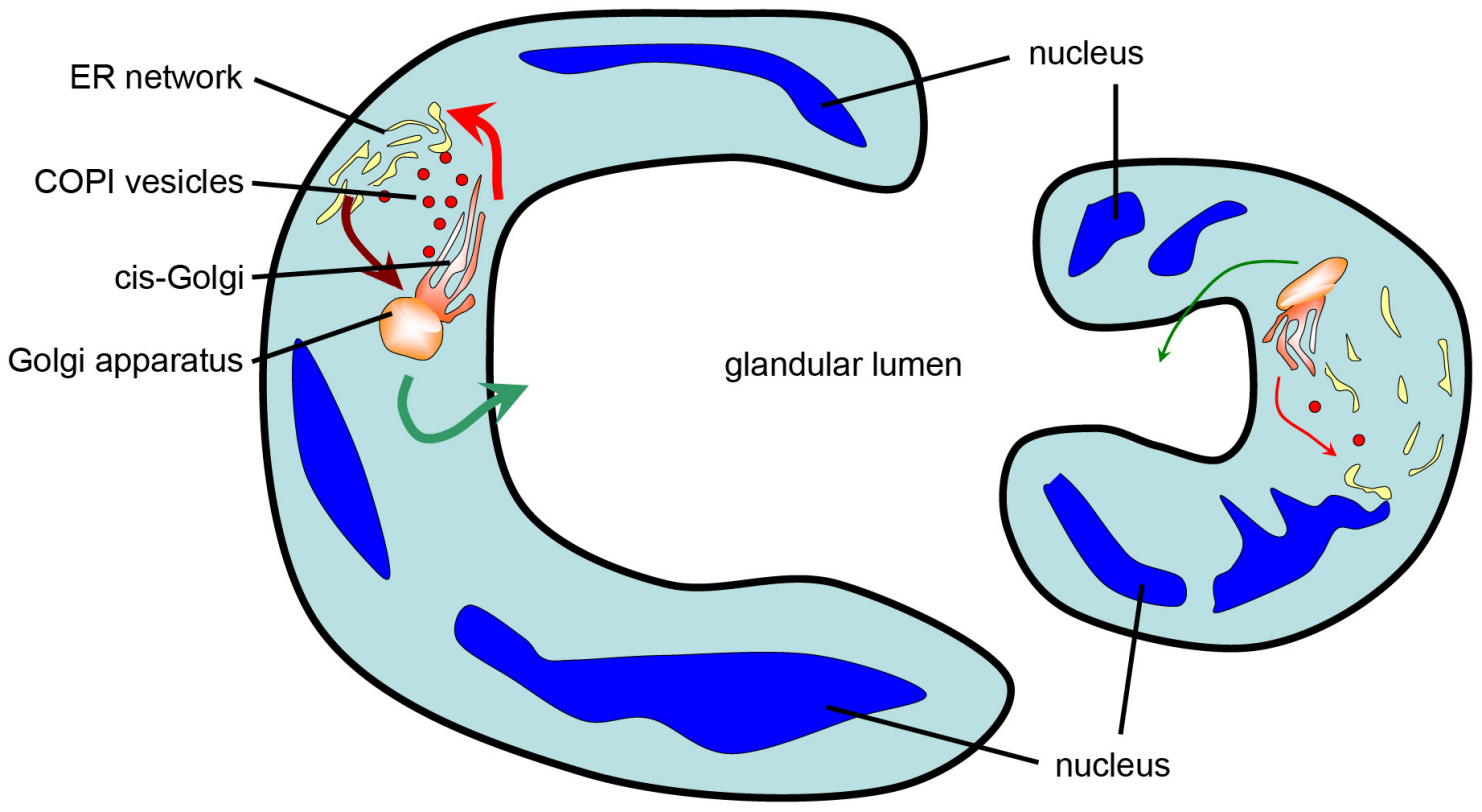
Schematic illustration of PSG cross-section in wild type and COPI-knockdown silkworm. In wild type (left), efficient COPI retrograde transport (thicker red arrow) and ER-to-Golgi anterograde transport (brown arrow) maintain the integrity of the endomembrane system and abundant luminal protein secretion (thicker green arrow). The accumulating luminal matrix generates a distending force to expand tube diameter. In COPI-knockdown silkworm (right), the disruption of COPI transport (thinner red arrow) leads to structural defects of the endomembrane system and subsequent inefficient secretion (thinner green arrow), causing a failure in the tube expansion.

Here, we demonstrated that the absence of silkworm COPI led to tube expansion deficiency of the PSG, and further established that COPI deficiency disrupts the integrity of the Golgi apparatus. COPI vesicles play a well-established role in the Golgi-to-ER retrograde transport [21], [22], and may also transport anterogradely between different cisterns of the Golgi apparatus [13], [23], or be associated with endosome-related activities [24], [25]. Therefore, the COPI defects interrupt the Golgi-ER communication, widely disrupt protein shuttling in silkworm PSG, and dramatically affect the integrity and balance of endomembrane system (Figure 9).

It is reported previously that the structural defects of secretory apparatus (Golgi and ER) reduces the secretion of the luminal protein [40], [42]. Moreover, in *Drosophila* and zebrafish, the accumulating luminal matrix or fluid generates a distending force to expand the tube diameter [43], [44]. Here, we provided evidence that the PSG tube expansion deficiency in COPI knockdown silkworms was due to the abnormal and disrupted endomembrane system. In COPI-knockdown PSG, the intracellular transport is interrupted, disrupting the integrity of endomembrane system, suppressing the luminal matrix secretion, and leading to a subsequent decrease of the distending force generated inside the PSG lumen. Considering that fibroin is the most abundant luminal matrix in silkworm PSG [4], we hypothesized that it is the significant reduction of the secreted fibroin that results in the dropped distending force. Therefore, the PSG lumen fails to expand in the COPI-knockdown PSG (Figure 9).

A fibroin secretion-deficient silkworm mutant, *Nd-s^D^*, is previously reported to possess an immature PSG [45], which is similar to the phenotype in COPI-knockdown silkworm. It is also due to the dramatic decrease of the secreted fibroin, we speculate, that results in the reduced distending force and following narrower PSG lumen.

Most reports suggest that COPI subunits exhibit highly conserved localization and physiological function. Although this study of silkworm COPI suggests its conservation throughout evolution, some of our results revealed some surprising differences between the coatomer subunits of silkworm and other organisms. First, the sequence of Bm-β-COP identified by RT-PCR lacks 400 amino acids compared with its homologues in other organisms. However, the predicted β-COP cDNA sequence from the silkworm genome contains the lost 400 amino acids (data not shown), suggesting our identified β-COP may be produced by alternative splicing. Considering that no splicing variant of coatomers has been reported in any organisms, the silkworm β-COP isoforms with or without the 400 amino acids seem worth further investigation. Second, we noted different expression levels of coatomer subunits in silkworm brain (Figure 2A–F, left), with lower mRNA levels for α, β, and β′-COP (Figure 2A–C, left), but higher for δ, ε, and ζ-COP (Figure 2D–F, left). Therefore, it is logical to speculate that δ, ε, and ζ subunits have a specific role in silkworm brain, which also needs further investigation.

The silkworm PSG may have several advantages for studying intracellular transport [2]. The PSG tubule, surrounded by two PSG cells, is responsible for silk production and secretion [5], [8]. In the PSG cells, there exists an extensive endomembrane system, including Golgi apparatus, ER, and secretory granules [41]. It is likely that COPI-mediated intracellular transport may be abundant in this highly differentiated secretory tissue compared with other tissues. Meanwhile, PSGs are easily dissected for biochemical analysis; silkworm genomic sequences and the cDNA database are available [1], [31], [32]; RNAi technology established in silkworm recently [27], in combination with the BmNPV baculovirus transfection system [2], transgenic silkworm technique [6], and micromanipulation tools [46], offer researchers a great opportunity to assess the physiological functions of COPI vesicles. Moreover, many coat proteins have still not been identified in silkworm, such as COPII coats [10], [14] and adaptors for clathrin coats [47]. These coat proteins, in combination with COPI coats, envelop the transported vesicles, and form an interconnected network to capture different types of cargoes. We believe that identifying more vesicle-associated coat proteins and more coatomer isoform would further advance our understanding of the intracellular vesicle transport in silkworm PSG.

Although much progress has been made on elucidating COPI vesicle formation, the mechanism of COPI transport is still elusive and controversial [48]. Kinesin-1 is reported to participate in COPI transport [49], but some data suggest the dispensability of Kinesin-1 in COPI movement [50]. Recently, Kinesin-2 is reported to participate in the COPI-dependent Golgi-to-ER retrograde transport [51]. Therefore, with the help of identification of silkworm COPI complex, we will investigate the molecular mechanism of vesicle transport in silkworm PSG. In PSG cells, whether COPI vesicles are transported by BmKinesin-1 or other motor proteins? What are the cargoes transported by COPI vesicles in PSG cells? Could we identify any novel proteins involved in COPI formation or transport? Future answers to these questions may deepen and broaden our understandings of COPI-related processes in silkworm and in mammals.

## Materials and Methods

### *Bombyx mori* strain

The embryos of *B. mori* strain (p50) were hatched and reared as previously described [2], and the artificial diet was provided by the Chinese Academy of Agricultural Sciences.

### Bioinformatic analysis

Sequences for cloning analysis were obtained from http://silkworm.genomics.org.cn/ [31] and http://papilio.ab.a.u-tokyo.ac.jp/silkbase/ [32]. The amino acid sequences of coatomers in organisms were derived from NCBI, and were aligned using MAFFT [52]. The neighbor-joining trees were inferred and decorated by MEGA 3.1 [53].

### RNA isolation and cDNA cloning

The RNA was isolated and reverse-transcripted according to previous reports [34]. Six silkworm coatomer subunits, α-, β-, β′-, δ-, ε-, and ζ-COP, were amplified (30 cycles of 94°C for 30 s and 60°C for 30 s and 72°C for 3 min) using LA Taq DNA polymerase (Takara) (see Table S2 for primer information). Then, the PCR products were cloned into pCR2.1 vector (Invitrogen) and were delivered for sequencing (Invitrogen). To rule out errors introduced by PCR, we delivered at least three clones of each band for sequencing.

### Real-Time quantitative PCR

Real-time quantitative PCR was performed by an ABI 7300 Detection System (Applied Biosystems) using the SYBR Green PCR Master Mix (Applied Biosystems) as previously described [34]. *Ribosomal protein L3* (*RpL3*) was served as a reference control [33] and the 2^−ΔΔCT^ method [54] was used (see Table S2 for primer information).

### Antibody production

Regions (amino acids 1021–1240 for α-COP; 221–400 for β′-COP; 251–500 for γ-COP) of silkworm α-, β′-, and γ-COP were amplified and cloned into the *Eco*RI/*Xho*I site in the expression vector pGEX-6P-1, respectively (see Table S2 for primer information). The fusion proteins were collected and purified as previously described [34], and were used as antigens to immunize both rabbits and mice.

### Immunoblotting analysis

After gel separation by SDS-PAGE, the proteins were transferred onto PVDF membranes (Millipore) in a semidry transfer cell (Bio-Rad). The membranes were blocked and subsequently probed with primary antibodies at 4°C overnight. HRP-conjugated goat anti-mouse/rabbit IgG (Jackson ImmunoResearch Laboratories, Inc.) were used as secondary antibodies. Primary antibodies used were mouse anti-α-COP polyclonal antibody, anti-β′-COP polyclonal antibody, anti-γ-COP polyclonal antibody, anti-α-tubulin monoclonal antibody (B-5-1-2, Sigma), rabbit anti-α-COP polyclonal antibody, and rabbit anti-β′-COP polyclonal antibody.

### Transfection and immunofluorescence

The δ-, ε-, ζ-COP were cloned into the *Bam*HI/*Xho*I site of the pFastBac-1-based pFastBac-hr5/IE1-GFP vector [34] (see Table S2 for primer information). Vector pFastBac-hr5/IE1 was constructed with both IE-1 promoter [37] and hr5 enhancer [38] sequentially inserted into pFastBac-1 vector (Invitrogen) between *Sna*BI and *Bam*HI.

BmN cells were maintained as in previous reports [34] and transfected by Cellfectin II Reagent (Invitrogen). Cells were fixed 24–48 hours post-transfection, and then subjected to immunofluorescence staining. After permeabilization and blocking [34], the cells were incubated with primary antibodies (anti-α-COP polyclonal antibody, mouse anti-Myc monoclonal antibody, 9E10 Upstate, and/or rabbit anti-GM130 polyclonal antibody, Abcam) and secondary antibodies (Alexa Fluor 488- and/or 568-conjugated goat anti-mouse and/or anti-rabbit IgG, Molecular Probes). The samples were observed under a TCS SP2 confocal microscope (Leica) equipped with a 100×/1.4 numerical aperture oil-immersion objective lens.

For immunofluorescence of cryosections, the whole third-instar larvae or the dissected PSGs were fixed and prepared for sectioning on a Leica cryostat CM1850. This sectioning and immunofluorescence process has been described previously [34]. The primary antibodies used were mouse/rabbit anti-α-COP polyclonal antibody, rabbit anti-GM130 polyclonal antibody (Abcam), and mouse anti-tubulin monoclonal antibody (Sigma). Samples were subsequently observed under a confocal microscope (Leica).

### Electron microscopy

For electron microscopy, we fixed the dissected PSGs and processed the samples as previously reported [55]. For immunoelectron microscopy, whole third-instar larvae were fixed in 3% paraformaldehyde in PBS containing 0.1% glutaraldehyde and 4% sucrose. After gradient dehydration in 30–100% methanol, the samples were infiltrated by resin (Lowicryl K4M) at −20°C and embedded in capsules fully filled with resin. The embedded samples were polymerized under UV light, followed by ultrathin section preparation and post-embedding immunolabeling. The primary antibodies used were mouse anti-α-COP polyclonal antibody and rabbit anti-GM130 polyclonal antibody (Abcam). The secondary antibodies were 5 nm or 10 nm gold-conjugated goat anti-mouse/rabbit IgG (Sigma).

### DsRNA synthesis and injection

The templates for dsRNA synthesis were amplified by PCR using gene-specific primers containing T7 polymerase sites as previously described [26], [27] (see Table S2 for primer information). DsRNAs were synthesized using a MEGAscript Kit (Ambion) and extracted according to the manufacturer's instructions. In detail, the dsRNA of α-COP corresponds to nucleotides 13–660 and 3061–3720; the dsRNA of β′-COP corresponds to nucleotides 1–660 and 661–1200; the dsRNA of γ-COP corresponds to nucleotides 751–1500 base pair; and GFP dsRNA served as a control. The concentrations of dsRNAs diluted in DEPC-treated H_2_O were measured. Based on previous reports [56], [57], 1 µg dsRNAs were respectively injected into larval hemolymph using pulled-glass capillary needles. Five days after injection, the silkglands were dissected out. After being photographed under a microscope (IX71 inverted fluorescence microscope, Olympus), the PSGs were homogenized for Western blotting analysis, or were fixed for frozen sectioning and electron microscopy. The amounts of coatomers and tubulin were estimated by Western blotting analysis. From the snapshots of PSG, we measured the luminal diameters of the PSGs from more than eight silkworms at similar sites (approx. 200 µm from the curve between MSG and PSG).

## Supporting Information

Figure S1 Production of silkworm coatomer antibody. Western blotting analysis showed that produced mouse and rabbit polyclonal antibodies of α-COP, β′-COP, and γ-COP could detect specific bands.
(3.27 MB TIF)

Table S1 The detailed information of seven silkworm coatomers. The gene name, accession number, nucleotide/amino acid length, predicted molecular weight, and identities between different organisms are listed in this table.
(8.40 MB TIF)

Table S2 The primers we used.
(0.11 MB PDF)
