# Supplementary figures and images for: Silkworm Coatomers and Their Role in Tube Expansion of Posterior Silkgland
Source: PLoS One. 2010 Oct 12;5(10):e13252. doi: 10.1371/journal.pone.0013252 (PMC2953498; doi:10.1371/journal.pone.0013252)

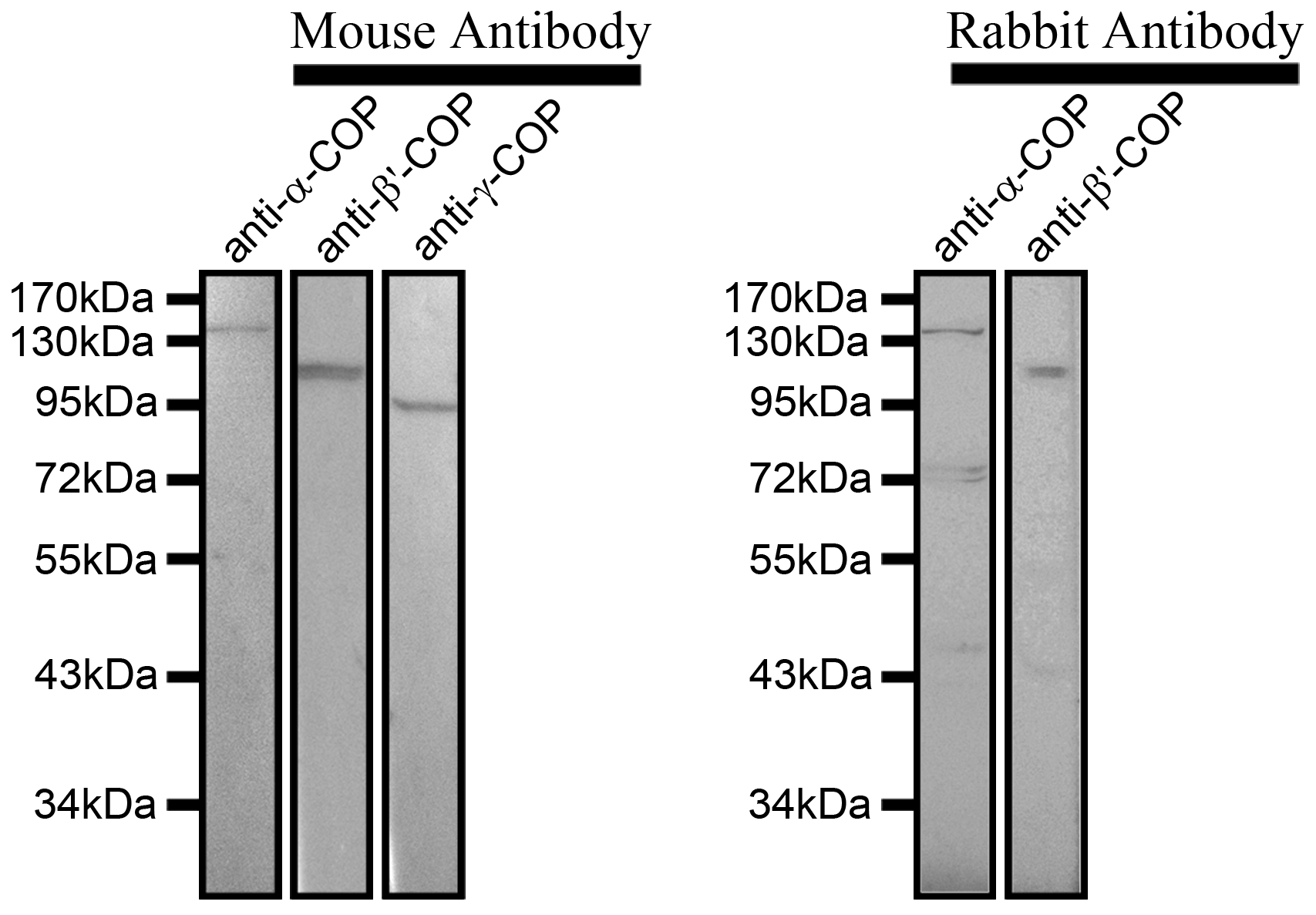

Supplement: Figure S1 — Production of silkworm coatomer antibody. Western blotting analysis showed that produced mouse and rabbit polyclonal antibodies of α-COP, β′-COP, and γ-COP could detect specific bands. (3.27 MB TIF) [file pone.0013252.s001.tif]

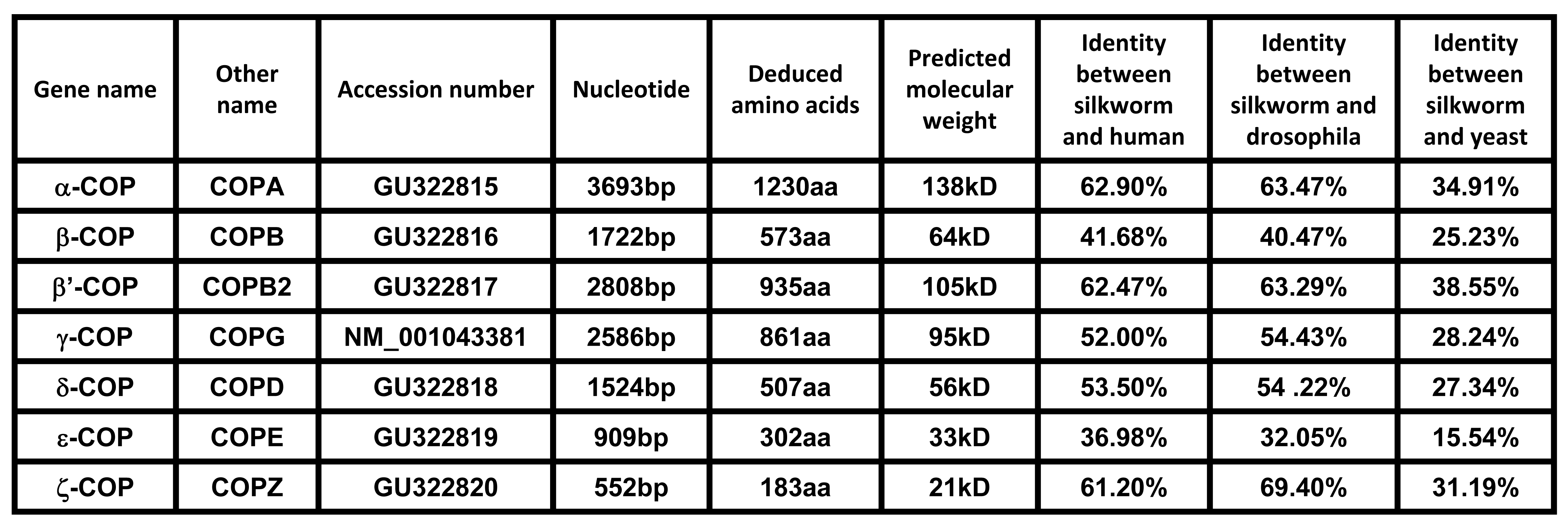

Supplement: Table S1 — The detailed information of seven silkworm coatomers. The gene name, accession number, nucleotide/amino acid length, predicted molecular weight, and identities between different organisms are listed in this table. (8.40 MB TIF) [file pone.0013252.s002.tif]
