# Supplementary material for: Silkworm Coatomers and Their Role in Tube Expansion of Posterior Silkgland
Source: PLoS One. 2010 Oct 12;5(10):e13252. doi: 10.1371/journal.pone.0013252 (PMC2953498; doi:10.1371/journal.pone.0013252)
Supplement: Table S2 — The primers we used. (0.11 MB PDF) [file pone.0013252.s003.pdf]

| **cDNA Cloning** | |
|---|---|
| α-COP | F-α-COP-ATG TTA AAA AAA TTC GAG ACG AAG TCT |
| | R-α-COP-CTA TCT GTT GAA CTG TAA GGG ACA |
| β-COP | F-β-COP-ATG GCG GTT GTG GAG CAG CCAT |
| | R-β-COP-TCA GGC GGC CGG CGT GGGG |
| β’-COP | F-β’-COP-ATG CCT CTG CGT CTG GAT ATC AA |
| | R-β’-COP-TTA GCC GAT TAA GTC TTC TAC GTC |
| δ-COP | F-δ-COP-ATG GTA CTT ATA GCC GCT ACA GTTT |
| | R-δ-COP-TTA AAC AAA CTC GTA TTT GTC TGC GT |
| ε-COP | F-ε-COP-ATG GCA CGT CAT CAG CAA GAT GTT |
| | R-ε-COP-TTA ACT AGC CAC TGA GGG TTT GTA |
| ζ-COP | F-ζ-COP-ATG GTT CAC ACA ACG ATG GAA GGT |
| | R-ζ-COP-TCA TTT CAA TAA GGA CCA TTT AAG TTG |

| **Real-time PCR** | |
|---|---|
| α-COP | F-RT-α-COP-CAG GCT GAT GCT GTT GTG AAG |
| | R-RT-α-COP–TAG GAT GAA AAC AGG CCC AGTT |
| β-COP | F-RT-β-COP-TGC AAG CTG AAA GAG CCT GAA |
| | R-RT-β-COP-GCT CGA GAC AGG CCC GTAT |
| β’-COP | F-RT-β’-COP-GAT CCT GCT GAC CGG TTC TG |
| | R-RT-β’-COP-GGT GCC TGC GTG CCA TAT |
| δ-COP | F-RT-δ-COP-GGC CAC TAC CAA CGA TGA CA |
| | R-RT-δ-COP-TCG GAC GGC CAA CAG TTT AC |
| ε-COP | F-RT-ε-COP-AGC AAG CTA TTA ATG AGG CAC AAA |
| | R-RT-ε-COP-GCA TCG CGT TGG AGT GCTA |
| ζ-COP | F-RT-ζ-COP-ACG GCG GTG TGA TAT TGG AT |
| | R-RT-ζ-COP-GCG CTG CCC GGC TTA |

| **Antibody Production** | |
|---|---|
| α-COP | F-α-COP-*Eco*RI-AAA GAA TTC TTG AAG CTA AGC GAC TTG GTG AGT |
| | R-α-COP-*Xho*I-AAA CTC GAG GGG ACA GAT ACG GAG GCC GAG |
| β’-COP | F-β’-COP-*Eco*RI-AAA GAA TTC TGT GTA CAA ACA TTG GAA GGT CATG |
| | R-β’-COP-*Xho*I-AAA CTC GAG GAA TGC CCA TAC AAA CTC CTG GG |
| γ-COP | F-γ-COP-*Eco*RI-GGG GAA TTC ATT GAA TCC TGT CTA CGCA |
| | R-γ-COP-*Xho*I-GGG CTC GAG CAA CTC AGG ACG GGA GGC TCC |

| **Vector Construction** | |
|---|---|
| δ-COP | F-δ-COP-*Bam*HI-CCC GGA TCC ATG GTA CTT ATA GCC GCT ACA GTTT |
| | R-δ-COP-*Xho*I-CCC CTC GAG AAC AAA CTC GTA TTT GTC TGC GTGG |
| | R-δ-COP-[Myc]-*Xho*I-CCC CTC GAG TCA [CAG ATC CTC TTC AGA GAT GAG TTT CTG CTC] AAC |

| | |
|---|---|
| | AAA CTC GTA TTT GTC TGC GTGG |
| ε-COP | F-ε-COP-*Bam*HI-CCC GGA TCC ATG GCA CGT CAT CAG CAA GAT GTT |
| | R-ε-COP-*Sal*I-CCC GTC GAC ACT AGC CAC TGA GGG TTT GTA CTG |

| **RNAi Experiment** | |
|---|---|
| α-COP | F-α-COP-I-[T7]- [GAA TTA ATA CGA CTC ACT ATA GGG AGA CCAC] TTC GAG ACG AAG TCT GCG CGG |
| | R-α-COP-I-[T7]- [GAA TTA ATA CGA CTC ACT ATA GGG AGA CCAC] TGG TAA AGT AGG ATG AAA ACA GGC |
| | F-α-COP-II-[T7]- [GAA TTA ATA CGA CTC ACT ATA GGG AGA CCAC] TTG AAG CTA AGC GAC TTG GTG AGT |
| | R-α-COP-II-[T7]- [GAA TTA ATA CGA CTC ACT ATA GGG AGA CCAC] GGG ACA GAT ACG GAG GCC GAG |
| β’-COP | F-β’-COP-I-[T7]- [GAA TTA ATA CGA CTC ACT ATA GGG AGA CCAC] ATG CCT CTG CGT CTG GAT ATC AA |
| | R-β’-COP-I-[T7]- [GAA TTA ATA CGA CTC ACT ATA GGG AGA CCAC] TGT CTT ATT TTG GTA ATC CCA TAT TTT |
| | F-β’-COP-II-[T7]- [GAA TTA ATA CGA CTC ACT ATA GGG AGA CCAC] TGT GTA CAA ACA TTG GAA GGT CATG |
| | R-β’-COP-II-[T7]- [GAA TTA ATA CGA CTC ACT ATA GGG AGA CCAC] GAA TGC CCA TAC AAA CTC CTG GG |
| γ-COP | F-γ-COP-[T7]- [GAA TTA ATA CGA CTC ACT ATA GGG AGA CCAC] GAA TTC ATT GAA TCC TGT CTA CGCA |
| | R-γ-COP-[T7]- [GAA TTA ATA CGA CTC ACT ATA GGG AGA CCAC] CAA CTC AGG ACG GGA GGC TCC |
